# Exosome secretion from hypoxic cancer cells reshapes the tumor microenvironment and mediates drug resistance

**DOI:** 10.20517/cdr.2022.38

**Published:** 2022-06-21

**Authors:** Kenneth K. W. To, William C. S. Cho

**Affiliations:** ^1^School of Pharmacy, Faculty of Medicine, The Chinese University of Hong Kong, Hong Kong SAR, China.; ^2^Department of Clinical Oncology, Queen Elizabeth Hospital, Hong Kong SAR, China.

**Keywords:** Hypoxia, drug resistance, exosome, tumor microenvironment, immunotherapy, non-coding RNA

## Abstract

Hypoxia is a common phenomenon in solid tumors as the poorly organized tumor vasculature cannot fulfill the increasing oxygen demand of rapidly expanding tumors. Under hypoxia, tumor cells reshape their microenvironment to sustain survival, promote metastasis, and develop resistance to therapy. Exosomes are extracellular vesicles secreted by most eukaryotic cells, including tumor cells. They are enriched with a selective collection of nucleic acids and proteins from the originating cells to mediate cell-to-cell communication. Accumulating evidence suggests that exosomes derived from tumor cells play critical roles in modulating the tumor microenvironment (TME). Hypoxia is known to stimulate the secretion of exosomes from tumor cells, thereby promoting intercellular communication of hypoxic tumors with the surrounding stromal tissues. Exosome-mediated signaling pathways under hypoxic conditions have been reported to cause angiogenesis, invasion, metastasis, drug resistance, and immune escape. Recently, the programmed cell death ligand-1 (PD-L1) has been reported to reside as a transmembrane protein in tumor exosomes. Exosomal PD-L1 was shown to suppress T cell effector function in the TME and cause drug resistance to immune checkpoint therapy. This review provides an update about the pivotal role of tumor-derived exosomes in drug resistance to chemotherapy and immunotherapy, particularly under hypoxic conditions. Emerging strategies that target the exosomes in the hypoxic TME to enhance the antitumor efficacy are discussed.

## INTRODUCTION

Hypoxia is a well-known hallmark of solid tumors when the tumor vasculature cannot provide adequate oxygen to support the aggressive growth of rapidly expanding tumors. Preclinical studies demonstrated that hypoxia mediates resistance to various modalities of cancer therapy, including chemotherapy, radiotherapy, and immunotherapy^[1,2]^. Tumor hypoxia may also promote invasion and metastasis. Extensive evidence is also available from clinical investigations to suggest that highly hypoxic tumors are associated with treatment failure, increased incidence of distant metastases, and dismal disease-free and overall survival^[3]^.

Exosomes are a unique form of extracellular vesicles with endosomal origin and sizes ranging from 30 to 100 nm. They are secreted from diverse cell types upon the fusion of multivesicular bodies with the plasma membranes^[4]^. Exosomes mediate intercellular crosstalk by transferring mRNAs, microRNAs (miRNAs), and proteins from donor to recipient cells^[5,6]^. Cargoes loaded in exosomes are biologically active when taken up by the recipient cell, and they lead to various downstream functions^[7]^. Tumor-derived exosomes have been shown to facilitate the intercellular transfer of pro-tumorigenic factors in the tumor microenvironment (TME)^[8,9]^. They promote angiogenesis, invasion, and proliferation in recipient cells to support tumor growth and a pro-metastatic phenotype. In a recent proteome profiling study of exosomes derived from human primary and metastatic colorectal cancer cells, selective enrichment of metastatic factors and signaling pathway components was observed^[10]^. In glioma, exosomes have been reported to convey signals between the tumor and TME to facilitate bidirectional communication^[11]^. Hypoxia is known to stimulate the secretion of exosomes from tumor cells, thereby promoting cell-to-cell communication between the hypoxic tumors and the surrounding stromal tissues. Exosomal cargoes are also altered under hypoxic conditions to stimulate angiogenesis, invasion, metastasis, therapeutic resistance, and immune escape^[12]^.

In this article, we summarize the critical role played by hypoxic tumor-derived exosomes in tumor progression and resistance to cancer therapy. Novel approaches to target the exosomes in the hypoxic TME to potentiate the anticancer efficacy are discussed.

## HYPOXIC TME

TME is a dynamic and complex system around a tumor, which is composed of blood vessels, fibroblasts, extracellular matrix fibers, immune cells, and signaling molecules, supporting proliferation, metastasis, and therapy resistance of tumor cells^[13]^. The rapid proliferation of cancer cells and aberrant blood vessel formation create hypoxic conditions in malignant tumors. Hypoxia is considered a hallmark of TME, which is generally defined by a low oxygen tension of below 10 mmHg^[14]^. It is also known to orchestrate the various malignant phenotypes of cancer cells by activating multiple oncogenic signaling pathways. At the molecular level, the hypoxic TME is largely regulated by the hypoxia-inducible factor (HIF) family of transcriptional factors^[15]^. Accumulating evidence suggests that tumor-derived exosomes play a critical role in invigorating the interaction between cancerous and non-cancerous cells in the hypoxic TME to propel cancer progression^[16]^.

## EFFECT OF HYPOXIA ON TUMOR-DERIVED EXOSOME

### Induction of exosome release 

TME-associated cells have been shown to secrete more exosomes than normal cells to promote intercellular communication and nutrient exchange^[17]^. In the clinical setting, the number of circulating exosomes collected from the blood of cancer patients was estimated to be more than two-fold higher than that of healthy subjects^[18]^. Granulocytic myeloid-derived suppressor cells (G-MDSCs) in tumors were reported to produce more exosomes than those found in the spleen^[19]^. Indeed, more secretion of exosomes from cancer cells has been reported in breast cancer^[20]^, colorectal cancer^[21]^, gastric cancer^[22]^, glioma^[23]^, hepatocellular carcinoma^[24]^, and pancreatic cancer^[25]^. Interestingly and in contrast, hyperoxia (excessive oxygen tension) has been reported to reduce the number of exosomes released from colorectal cancer cells^[19]^. It is noteworthy that the induction of exosome secretion in hypoxia may be universal because more exosomes were also released from non-cancerous cells (including bone, cardiac, and kidney cells) under hypoxic conditions^[26-28]^. To date, the detailed mechanism leading to increased exosome release in hypoxia is still not clear. Nevertheless, two Rab GTPases (Rab27a and Rab27b) were shown to promote exosome secretion by facilitating the docking of the multivesicular bodies at the plasma membrane^[29]^, whereas Rab7 was reported to direct exosomes to lysosomes for degradation^[30]^. In ovarian cancer cells, hypoxia was shown to upregulate Rab27a but downregulate Rab7 to promote exosome release^[31]^.

### Increase in exosomal heterogeneity

Exosomes secreted from different cells contain different cargoes and markers. On the other hand, exosomes released from the same cell line can carry different constituents^[18]^. While this heterogeneity is advantageous for exosome applications, it poses obstacles to a thorough understanding of exosome biology^[32,33]^. Depending on the cell state, exosomes of different sizes and carrying different cargoes are secreted^[34]^. To this end, exosomes secreted from glioma cells were shown to reflect the hypoxic status, and they mediate hypoxia-dependent activation of neovascularization during tumor development^[34]^. The heterogeneous exosome population could have a different functional effect on the recipient cells^[35,36]^.

### Production of smaller exosome 

The size of exosomes released from mammalian cells is known to vary considerably in individual cells because of their unique exosome biogenesis process^[36]^. Interestingly, it has been reported that exosome size is associated with different disease states. In non-small cell lung cancer (NSCLC) patients, a smaller exosome detected in the pulmonary vein is associated with a shorter time to relapse and shorter overall survival after curative surgery^[37]^. It is generally believed that hypoxia tends to release smaller exosomes. Relatively smaller exosomes have been reported in different cancer cell lines, including colon^[21]^, pancreatic^[25]^, and prostate^[38]^ cancer cells. Under hypoxic conditions, it is believed that the supply of cellular materials for membrane synthesis may not meet the demand for exosome production, thus leading to the secretion of smaller exosomes^[12]^. It is also hypothesized that smaller exosomes could be transmitted more easily via the blood circulation to metastatic sites to form the pre-metastatic niches because hypoxia changes the hemodynamics in the tumor vasculature^[12]^. Moreover, smaller exosomes could be internalized faster and more efficiently than larger exosomes in the recipient cells^[39]^. An ongoing prospective clinical trial (NCT02310451) is underway, which examines exosome size and various other parameters as potential biomarkers in melanoma patients.

### Influence on exosomal cargo sorting 

It is known that exosome content is not simply reflecting the cellular content of its origin. Some sorting mechanisms are in place to facilitate specific cargo sorting processes. Hypoxia has been shown to affect the sorting of the following three major cargoes (i.e., nucleic acids, proteins, and lipids) into exosomes.

### Nucleic acids

The effect of hypoxia on the nucleic acid content in tumor-derived exosomes has recently been summarized in a few excellent reviews^[40,41]^. It is noteworthy that only limited studies have reported the effect of hypoxia on cancer-derived exosomal DNA. On the other hand, the alteration and biological effects of non-coding RNAs (ncRNAs) in tumor-secreted exosomes under hypoxia have been extensively studied. Representative examples are summarized in Table 1. Various mechanisms have been proposed to regulate the expression of ncRNAs in hypoxia. For the HIF-dependent mechanism, the binding of HIF-1α and/or HIF-2α to the hypoxic response element (HRE) of the gene promoter of miR-155 has been reported^[42,43]^. Moreover, miRNAs are differentially sorted into exosomes according to their specific sequence or motif^[44]^. A few RNA binding proteins, such as SYNCRIP, were shown to direct specific miRNAs sharing a common extra-seed sequence hEXO motif for enrichment in the exosomes^[45]^. Moreover, hypoxia also affects RNA alternative splicing^[46,47]^ and RNA editing^[48]^, which could make specific miRNAs more suitable for loading into tumor-derived exosomes. On the other hand, hypoxia was also reported to regulate a few facilitators which load nucleic acids into exosomes. The RNA binding proteins YBX1 and hnRNPA1 were reported to mediate the selective loading of miR-133 and miR-1246, respectively, into tumor-derived exosomes under hypoxia^[49,50]^.

**Table 1:** Representative non-coding RNA cargoes secreted in hypoxic cancer cell-derived exosomes to modulate the tumor microenvironment

**Non-coding RNA**	**Donor cell**	**Recipient cell**	**Biological function (mechanism)**	**Reference**
let7a	Melanoma	Macrophage	Induce M2 polarization of TAM and promote oxidative phosphorylation to support cancer growth (downregulation of insulin-AKT-mTOR signaling)	[121]
linc-RoR	Hepatocellular carcinoma	Hepatocellular carcinoma	Promote cancer cell proliferation; mediate chemoresistance (induction of PDK1 and HIF-1α protein expression by suppressing miR-145)	[78]
lncRNA UCA1	Bladder cancer	Bladder cancer	Promote cancer growth; stimulate migration and invasion (induction of EMT)	[95]
miR-10a	Glioma	MDSC	Activate MDSC (regulation of RORA/IκBα/NF-κB signaling)	[113]
miR-21	Glioma	MDSC	Activate MDSC (regulation of PTEN/PI3K/AKT signaling)	[113]
miR-23a	Lung cancer	Endothelial cells	Promote angiogenesis; increase vascular permeability (inhibition of the propyl hydroxylases PHD1 and PHD2, thereby stabilizing HIF-1α protein; inhibition of the tight junction protein ZO-1)	[92]
miR-24-3p	Nasopharyngeal carcinoma	T cell	Inhibit T cell proliferation and differentiation (inhibition of FGF11; upregulation of p-ERK, p-STAT1, and p-STAT3)	[103]
miR-25-3p	Breast cancer	Breast cancer and macrophage	Promote cell proliferation and migration (stimulation of IL-6 secretion from macrophage via NF-κB signaling)	[94]
miR-125b-5p	Ovarian cancer	Macrophage	Induce M2 polarization (regulation of SOCS4/5-STAT3 pathway)	[120]
miR-210	BMSC	Lung cancer	Promote metastasis (induction of STAT3 to mediate EMT)	[73]
miR-301a-3p	Pancreatic cancer	Macrophage	Induce M2 polarization (downregulation of PTEN and activation of PI3K/Akt signaling)	[118]
miR-5100	BMSC	Lung cancer	Promote metastasis (induction of STAT3 to mediate EMT)	[97]

BMSC: Bone marrow-derived stem cell; EMT: epithelial-mesenchymal transition; linc-RoR: long intergenic non-protein coding; RNA: regulation of reprogramming; lncRNA: long non-coding RNA; MDSC: myeloid-derived suppressor cells; TAM: tumor-associated macrophage.

Hypoxia has been shown to regulate the expression of several ncRNAs, which affect the expression of HIF-1α and subsequently form positive or negative feedback loops to modulate the hypoxic TME^[51,52]^. In hypoxic gastric cancer cells, such a positive feedback loop involving miR-301a-3p, PHD3, and HIF-1α has been reported^[53]^. miR-301a-3p was upregulated in hypoxic gastric cancer cells and the tumor-secreted exosomes^[53]^. miR-301a-3p was subsequently shown to suppress the hydroxylase PHD3 and thus promote the protein stability of HIF-1α to maintain the hypoxic response. In pancreatic cancer, the hypoxic induction of a HIF-1α-stabilizing circular RNA (cirZNF91) in cancer-secreted exosomes was reported to promote chemoresistance in normoxic cancer cells^[54]^. Upon transmission to normoxic cells, circZNF91 was found to bind competitively to miR-23b-3p, subsequently abolishing the inhibition of miR-23b-3p on its target Sirtuin1 (SIRT1). The upregulated SIRT1 was shown to increase the deacetylation-dependent stability of HIF-1α protein, thus promoting glycolysis and chemoresistance in the recipient normoxic cancer cells^[54]^.

### Proteins

Similar to nucleic acids, proteins loaded into tumor-derived exosomes are not necessarily proportional to the cellular protein composition^[55]^. However, the precise mechanism contributing to specific exosomal protein sorting remains obscure. It has been postulated that hypoxia could influence the protein loading process of tumor-derived exosomes by regulating ubiquitination. Ubiquitinated proteins are recognized by ubiquitin-binding domains within multivesicular endosomes for degradation, thus limiting the amount of membrane available for exosome formation^[56]^. To this end, hypoxia is commonly known to control protein ubiquitination and ubiquitination-associated enzymes^[57]^.

Cell membrane glycoproteins are also known to participate in cell-to-cell and cell-environment communication^[58]^. Interestingly, the expression profile of glycoproteins in tumor-derived exosomes is different from that of healthy cells^[59]^. Moreover, glycoprotein expression could be affected by hypoxia^[60]^. As glycans are known to regulate protein sorting and uptake into exosomes, hypoxic cells have been shown to take up more exosomes in a proteoglycan-dependent manner^[61]^.

Proteins secreted in tumor exosomes are also known to participate in hypoxia-associated responses. In breast cancer, metastasis-associated protein 1-loaded exosomes were reported to transfer in between cancer cells to regulate the response to hypoxia and estrogen signaling^[62]^. Taken together, hypoxia promotes numerous malignant phenotypes of cancer cells by altering the exosome protein heterogeneity, whereas proteins in tumor-derived exosomes could contribute to the hypoxic response. Table 2 summarizes the representative exosomal protein cargoes and other constituents that are preferentially secreted from hypoxic tumors to module the TME.

**Table 2:** Exosomal protein cargoes and other constituents are preferentially secreted from hypoxic tumors to modulate the tumor microenvironment

**Cancer type**	**Type of cargo**	**Biological function and mechanism**	**Reference**
Colorectal cancer	Wnt4 protein	• Intercellular communication with normoxic cancer cells • Exosomal Wnt4 promoted the translocation of β-catenin to the nucleus in normoxic cells • Activation of β-catenin signaling enhanced cancer cell motility and invasion	[137]
Glioblastoma	TSP1, VEGF, LOX protein	• Promote cancer growth, angiogenesis, and metastasis	[138]
Glioblastoma	MMPs, IL-8, caveolin 1, PDGFs, and lysyl oxidase	• Induce angiogenesis *in vitro* and *ex vivo* through phenotypic modulation of endothelial cells • Stimulate endothelial cells to secrete potent growth factors and cytokines and to activate pericyte PI3K/Akt signaling to promote migration	[34]
Lung cancer	TGF-β1	• TGF-β1 downregulates NKG2D on the cell surface of NK cells to suppress NK cell cytotoxicity	[116]
Nasopharyngeal carcinoma	MMP13	• Promote migration and invasion	[139]
Nasopharyngeal carcinoma	HIF-1α	• Promote EMT to induce migration and invasion	[140]
Prostate cancer	Tetraspanins (CD63 and CD81), heat shock proteins (HSP90 and HSP70), and Annexin II	• Remodel the epithelial adherens junction pathway to enhance invasiveness and stemness of naïve prostate cancer cells	[38]
Prostate cancer	Lactic acid	• Under chronic hypoxia, prostate cancer cells secrete more exosomes as a survival mechanism to remove metabolic waste	[141]

IL-8; Interleukin-8; LOX: protein lysine 6-oxidase; MMP: matrix metalloproteinase; PDGF: platelet-derived growth factor; TSP1: thrombospondin-1; VEGF: vascular epithelial growth factor.

### Lipids

Various lipid components are important building blocks for exosome membranes. They include phosphatidylcholine, phosphatidylethanolamine, phosphatidylinositol, phosphatidylserine, phosphatidic acid, cholesterol, ceramide, sphingomyelin, and glycosphingolipids^[59]^. They play critical roles in the biogenesis of exosomes. Sphingomyelin is hydrolyzed to ceramide, which promotes the budding of multivesicular bodies from the endosomes^[63]^. On the other hand, ceramide is metabolized to sphingosine 1-phosphate, which interacts with the inhibitory G protein-coupled receptors to induce exosome biogenesis^[64]^. Interestingly, knockdown of a key cholesterol lipid efflux transporter ABCG1 was found to inhibit cancer growth, concomitantly with the intracellular accumulation of exosomes and the exosomal cargo^[65]^. Phosphatidylserine has been reported to play a critical role in facilitating the uptake of exosomes secreted from hypoxia-induced stem cells by human umbilical cord endothelial cells^[66]^. Under hypoxic conditions, triglyceride was found to accumulate in prostate cancer cells and the secreted exosomes. Importantly, these exosomes were shown to promote cancer proliferation and invasion following reoxygenation^[67]^. Hypoxia is known to upregulate ceramide expression, which is proposed to promote the exosome release^[68]^.

### Effect on exosome uptake by recipient cells

Little is known about the effect of hypoxia on the intercellular transfer and uptake of tumor-derived exosomes from other components within the TME. A hypoxic environment is known to promote glycolysis and lactic acid production. Excess production of lactic acid results in acidic pH in the hypoxic TME. Given that an acidic environment is more suitable for the stability and isolation of exosomes^[69]^, the intracellular transport of exosomes may benefit from a hypoxic and acidic TME. It has also been proposed that the uptake of cancer-secreted exosomes by the recipient cells was found to be more efficient under hypoxia^[70]^. The smaller exosomes secreted from hypoxic tumors, as described above, may facilitate the more efficient intercellular transfer. However, this hypothesis has not been verified by a detailed experimental investigation. On the other hand, hypoxic tumor cells are also known to take up more exosomes from the surroundings^[61]^. Upon the recognition and binding of exosomes to recipient cells, the former is internalized by various processes, including endocytosis via clathrin, caveolae, or lipid raft-dependent manner. Under hypoxic conditions and the closely associated acidic cellular environment, exosome uptake could be promoted by lipid raft-dependent endocytosis^[61]^, caveolin-dependent endocytosis^[71]^, and phagocytosis^[72]^.

## REGULATION OF TME BY HYPOXIC TUMOR-DERIVED EXOSOMES

**Figure 1:**
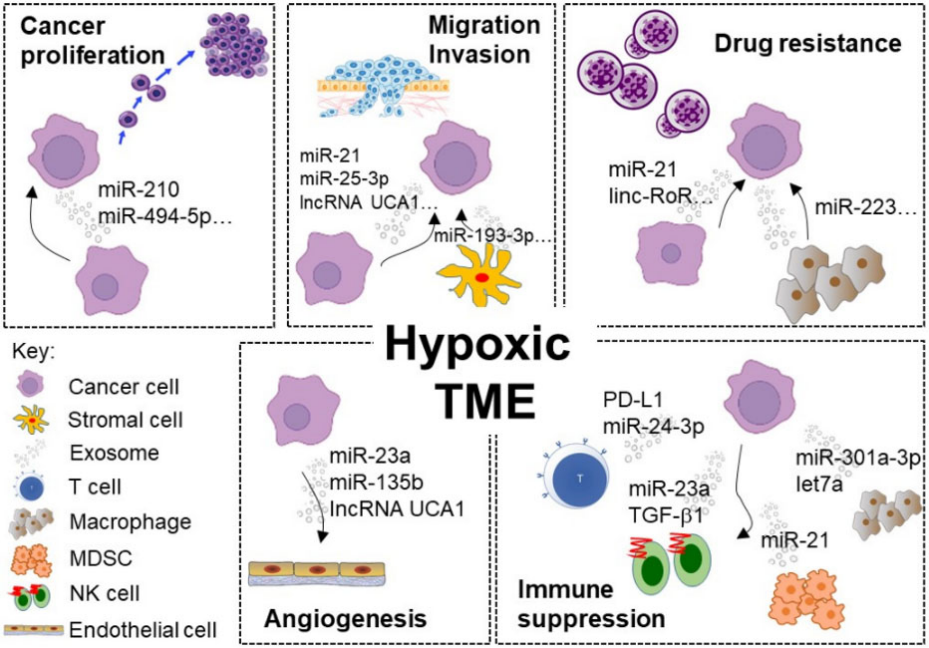
Representative examples of exosome-mediated intercellular communication within the hypoxic TME driving cancer progression, chemoresistance, and immune suppression. (1) Cancer proliferation: Hypoxic cancer-secreted exosomes were enriched with miRNAs supporting cell survival (e.g., miR-210) in the neighboring cancer cells. (2) Drug resistance: Numerous ncRNAs (e.g., linc-RoR and miR-21) were transferred via exosomes from hypoxic and resistant cancer cells to sensitive cells and induced drug resistance. (3) Migration and invasion: exosomes containing various ncRNAs (including lncRNA UCA1 and miR-193-3p) facilitate cancer–cancer or cancer–stromal intercellular communication to stimulate migration and invasion by modulating EMT. (4) Angiogenesis: ncRNAs (including miR-23a and lncRNA UCA1) were enriched in the exosomes secreted from hypoxic tumor cells to promote tumor vascular endothelial cell proliferation and angiogenesis in HIF-1α-dependent or -independent pathway. (5) Immune suppression: Exosomes enriched with miRNAs (e.g., miR-23a and let-7a) and other immunosuppressive molecules (e.g., PD-L1 and TGF-β1) were secreted from hypoxic tumors to promote an immunosuppressive TME. TME: Tumor microenvironment.

As described in the previous section, hypoxia significantly alters the properties of exosomes secreted from cancer cells. Accumulating evidence indicates that the altered tumor exosomes are responsible for the reshaping of TME, thereby promoting cancer cell proliferation, chemoresistance, metastasis, and angiogenesis [Figure 1].

### Promotion of cancer proliferation and chemoresistance by hypoxic tumor-derived exosomes

Within the hypoxic TME, cancer cells are known to secrete pro-tumorigenic molecules in the exosomes to promote cancer survival and proliferation. miR-210 is one of the most extensively studied hypoxia-induced miRNAs driving cancer progression^[73]^. In breast cancer, the abundance of miR-210 was reported to be remarkably higher in the exosomes derived from hypoxic cancer cells than those from normoxic ones, which allows the cells to sustain survival under hypoxia^[74]^. A set of differentially expressed exosomal miRNAs has been identified in the exosomes secreted from patient-derived melanoma cells under hypoxic culture conditions^[75]^. Hypoxia was found to upregulate miR-494-5p, miR-4497, miR-513a-5p, and miR-6087 but downregulate miR-125b-5p, miR-21-5p, and miR-3934-5p in the exosome^[75]^. Interestingly, the alteration of miRNAs was closely associated with cancer survival according to bioinformatics pathway analysis^[75]^. Exosomes secreted by human glioma were also shown to promote the differentiation of neural stem cells into astrocytes^[76]^. Transcripts related to cell proliferation and astrocyte differentiation were found to be remarkably upregulated in human mesenchymal stem cells when co-cultured with glioma-secreted exosomes^[76]^. Hypoxic tumor-secreted exosomes may represent an important therapeutic target that mediates the aggressiveness of glioma.

Hypoxia is known to mediate chemoresistance by regulating the cell cycle, autophagy, cell senescence, and drug efflux transporters. In recent years, the emerging role of hypoxic cancer cell-derived exosomes in reshaping the TME and causing chemoresistance is also revealed. In NSCLC, hypoxic cancer-derived exosomes have been shown to induce cisplatin resistance in normoxic cancer cells through the transmission of miR-21^[77]^. The transfer of miR-21 from hypoxic cell-derived exosome to normoxic cancer cells was demonstrated to downregulate PTEN and the PI3K/Akt pathway, which subsequently induced cisplatin resistance^[77]^. In hepatocellular carcinoma, the abundance of a stress-responsive lncRNA (linc-RoR) was significantly increased in hypoxic cancer-derived exosomes than in their normoxic counterpart^[78]^, which is associated with resistance to sorafenib and doxorubicin. Linc-RoR was shown to induce TGF-β, thereby suppressing chemotherapy-induced cell death but promoting tumor-initiating cell proliferation^[78]^. Stromal cells, such as cancer-associated fibroblasts (CAFs) in the TME, could also mediate chemoresistance in cancer cells. miR-223 was upregulated in TAMs and TAM-derived exosomes under hypoxia^[79]^. miR-223 loaded in hypoxic exosomes was shown to reduce apoptosis and induce drug resistance in ovarian cancer by downregulating PTEN and thus activating PI3K/Akt signaling^[79]^.

Moreover, exosomes have also been shown to mediate the transfer of the drug-resistant phenotype. Drug-sensitive cancer cells have been shown to become drug-resistant following the incorporation of exosomes shed from drug-resistant cancer cells^[80-84]^. Moreover, exosomes were shown to be involved in the intercellular transfer of functional ABCB1 (P-gp) from multidrug-resistant donor cells to drug-sensitive recipient cells^[81,85-87]^. Furthermore, exosomes have also been reported to mediate drug resistance by exporting specific drugs via the exosome pathway^[88]^ and neutralizing antibody-based chemotherapy^[89]^.

### Induction of cancer angiogenesis by hypoxic tumor-derived exosomes 

The induction of angiogenesis by hypoxia has been extensively studied^[90]^. More recently, accumulating evidence demonstrates that hypoxic tumor-derived exosomes played a significant role in angiogenesis. In malignant glioblastoma multiforme, exosomes derived from cancer cells under hypoxia were shown to induce angiogenesis by stimulating cytokine and growth factor secretion from endothelial cells, subsequently promoting pericyte migration^[34]^. In pancreatic cancer, the exosomal lncRNA UCA1 secreted from cancer cells under hypoxic conditions was shown to promote angiogenesis via a miR-96-5p/AMOTL2/ERK1/2 pathway^[91]^. In lung cancer, miR-23a secreted in tumor-derived exosomes in hypoxia was reported to target the key HIF-1α regulators (propyl hydroxylases PHD1 and PHD2), thereby sustaining the overexpression of HIF-1α and promoting angiogenesis^[92]^. Moreover, hypoxia-induced exosomal miR-23a was also shown to inhibit the tight junction protein ZO-1 and increase vascular permeability^[92]^. It is noteworthy that most studies in this research area were conducted under acute hypoxic conditions. Umezu *et al*. were the first to report intercellular communication via exosome under chronic hypoxia^[93]^. A few hypoxia-resistant multiple myeloma (MM) cell lines were developed after incubation in hypoxic conditions for more than six months to mimic the hypoxic bone marrow environment *in vivo*^[93]^. Increased exosomal level of miR-135b was detected in these hypoxia-resistant MM cells, and it was shown to promote endothelial tube formation under hypoxia via the HIF-FIH signaling pathway^[93]^.

### Promotion of cancer cell invasion and metastasis by hypoxic tumor-derived exosomes

Exosomal ncRNAs within the hypoxic TME are known to regulate tumor invasion and metastasis by modulating EMT. In breast cancer, higher expression of miR-25-3p in hypoxic cancer-derived exosomes was found to stimulate cancer proliferation and migration by inducing IL-6 secretion and activating NF-κB signaling in macrophages^[94]^. In bladder cancer, the lncRNA (UCA1) preferentially secreted by hypoxic cancer cells was shown to promote cancer growth by stimulating EMT both *in vitro* and *in vivo*^[95]^. In oral squamous cell carcinoma, a higher level of miR-21 was detected in the exosomes from hypoxic cancer than those from normoxic cancer to promote migration and invasion by inducing EMT^[96]^. The interaction between stromal and cancer cells via exosome within the TME plays a critical role in the initiation of metastasis. Lung cancer cells have been shown to take up exosomes secreted from hypoxic bone marrow-derived mesenchymal stem cells (BMSCs) and acquire a greater tendency for invasion^[97]^. Three miRNAs (miR-193-3p, miR-210-3p, and miR-5100) showing a high abundance in hypoxic BMSC-derived exosomes were transferred to cancer cells and subsequently activated STAT3 signaling to induce EMT in the lung cancer cells^[97]^. Interestingly, these three miRNAs were also found to be upregulated in plasma-derived exosomes from lung cancer patients with metastatic disease than in non-metastatic patients^[97]^.

### Modulation of cancer immune system by hypoxic tumor-derived exosomes 

**Figure 2:**
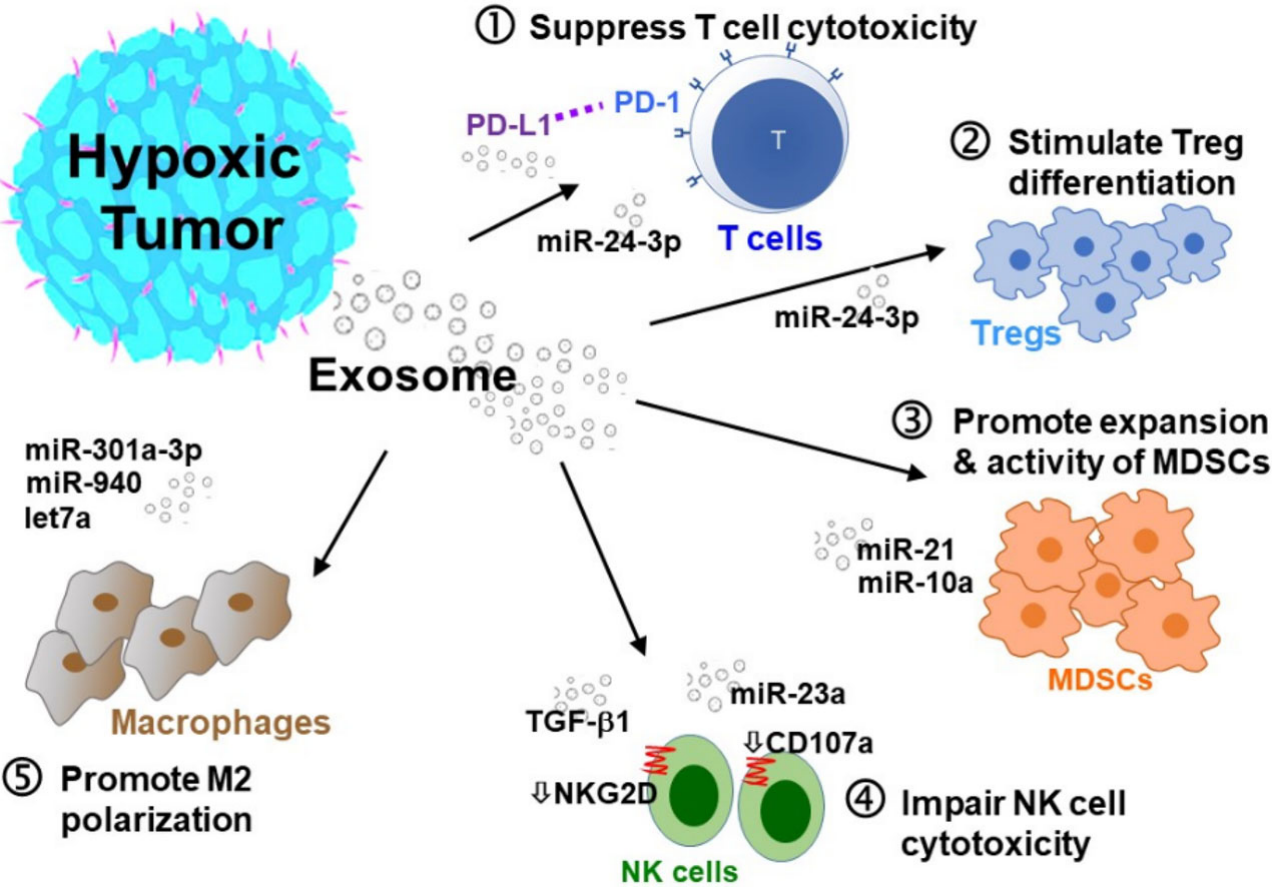
Hypoxic tumor-secreted exosomes promote an immunosuppressive TME. Hypoxic tumor-secreted exosomes promote an immunosuppressive TME by interfering with several intracellular pathways and modulating immune accessory cells, including cytotoxic T cells, T-regulatory cells (Tregs), myeloid-derived suppressor cells (MDSCs), natural killer cells (NK), and tumor-associated macrophages (TAMs). (1) Inhibition of T cell proliferation; (2) stimulation of Treg differentiation; (3) induction of MDSCs; (4) impairment of NK cells; and (5) stimulation of M2 polarization of TAMs.

Reduced immune surveillance is the major reason allowing primary tumors to develop metastasis in distant secondary organs^[98,99]^. Tumor-secreted exosomes have been reported to induce T-cell apoptosis, inhibit interferon gamma-dependent expression of macrophages, suppress natural killer (NK) cell activity, and increase myeloid-derived suppressor cell (MSDCs) population, which collectively suppress immune surveillance and allow tumor growth^[100,101]^. Figure 2 illustrates the major mechanisms by which hypoxic tumor-derived exosomes promote an immunosuppressive TME.

### Inhibition of T cell proliferation

Tumoral exosomes loaded with biologically active cargoes have been proposed to mediate the intercellular transmission of signals within the TME to promote immune escape and tumor progression. Ye *et al*. were the first to report a differential miRNA signature from nasopharyngeal carcinoma-derived exosomes to mediate T cell dysfunction^[102]^. The induction of exosomal miR-24-3p in nasopharyngeal carcinoma-derived exosomes under hypoxia was found to inhibit T cell proliferation but promote differentiation of T-regulatory cells (Tregs) by targeting FGF11 via the upregulation of p-ERK, p-STAT1, and p-STAT3 and downregulation of p-STAT5^[103]^.

Immune checkpoint inhibitors including anti-programmed cell death receptor (PD-1) (nivolumab and pembrolizumab) or anti-PD-ligand (PD-L1) (duralumab, atezolizumab, and avelumab) monoclonal antibodies are revolutionizing cancer therapy. They lead to durable anticancer responses and overall survival benefits in a wide range of cancer types^[104]^. PD-1 is an inhibitory receptor expressed on activated T cells, B cells, and natural killer cells, which blunt the immune response under physiological conditions. The T cell-mediated cancer-killing effect will be suppressed when PD-1 is occupied by its major ligand PD-L1 (expressed in tumor cells and infiltrating immune cells). Anti-PD-1/PD-L1 antibodies work by binding to the inhibitory PD-1 receptors on tumor-reactive T cells and PD-L1 on tumor cells, respectively, to disrupt the PD-1/PD-L1 interaction and reactivate the cytotoxic T cell activity.

Despite the breakthrough of anti-PD-1/PD-L1 immunotherapy, the response rate is low. Moreover, most patients who initially respond to immunotherapy will eventually relapse because of adaptive resistance. To maximize the full potential of anti-PD-1/PD-L1 immunotherapy, the mechanisms underlying these *de novo* and adaptive resistance mechanisms is a research area of intensive investigation. When T cells recognize the tumor antigen on the cancer surface, they release interferons to induce PD-L1 expression in cancer cells^[105]^. The increased PD-L1 expression in cancer cells will then lead to specific inhibition of T cell recognition of cancer, subsequently resulting in a phenomenon known as adaptive immune resistance and inhibiting the antitumor immune response. To this end, PD-L1 loaded in exosomes was shown to interact directly with T cells to suppress anticancer efficacy of chemotherapy in various cancer types, including breast^[106]^, gastric^[107]^ head and neck^[108]^, melanoma^[109]^, pancreatic^[110]^, and prostate^[111]^ cancer. It will be useful to elucidate whether hypoxia regulates the loading of PD-L1 into tumor-derived exosomes.

### Induction of MDSCs

MDSCs are a heterogeneous population of immune cells from the myeloid lineage which migrate to tumor sites to create an immunosuppressive TME^[112]^. MDSCs suppress adaptive and innate immunity by inhibiting T cell activation, promoting macrophage M2 polarization, inducing CAF differentiation, and inhibiting NK cell cytotoxicity. The abundance of MDSCs at tumor sites is known to correlate closely with poor clinical prognosis and reduce the efficacy of immunotherapy in cancer patients. In glioma, miR-21 and miR-10a secreted in tumor-derived exosomes under hypoxia have been reported to promote the expansion and activity of MDSCs *in vitro* and *in vivo* via the miR-21/PTEN/PI3K/AKT and miR-10a/RORA/IkBα/NF-κB pathways, respectively^[113]^. Therefore, novel strategies to modulate hypoxic tumor-secreted exosomes may be developed to regulate MDSCs and potentiate immunotherapy^[114]^. To this end, miR-21 loaded in γδ T cell-secreted exosomes has been shown to abate the function of MDSCs by targeting PTEN in a PD-L1-dependent manner^[115]^.

### Impairment of natural killer cells

Hypoxia is also known to promote an immunosuppressive TME by attenuating cytotoxic T cell and Impairment of natural killer (NK) cell-mediated tumor cell lysis. Berchem *et al*. were the first to report the secretion of non-coding RNAs in exosomes from hypoxic lung cancer cells to impair NK cell cytotoxicity^[116]^. Under hypoxic conditions, higher miR-23a expression was observed in lung cancer cell-derived exosomes, which impaired NK cell cytotoxicity by targeting CD107a^[116]^. Moreover, the hypoxic tumor-derived exosomes were also shown to transfer TGF-β1 to NK cells, thereby reducing the cell surface expression of the activating receptor NKG2D and inhibiting NK cell cytotoxicity^[116]^.

### Stimulation of M2 polarization of tumor-associated macrophages

TAMs refer to the major tumor-infiltrating immune cells, which interact with the tumors and tumor-associated macrophages (TME) to regulate tumor immunity^[117]^. Macrophage polarization is the process by which macrophages adopt distinct functional phenotypes in response to environmental stimuli and signals. M1 macrophages are functionally pro-inflammatory and antimicrobial, whereas M2 macrophages are anti-inflammatory. M1 and M2 macrophages exhibit a high degree of plasticity and are converted into each other upon changes within the TME or anticancer therapies. Under hypoxic pressure, tumor-derived exosomes have been shown to induce M2 polarization in various cancer types. In pancreatic cancer, miR-301a-3p was highly expressed in hypoxic cancer cell-derived exosomes, and it was shown to promote macrophage M2 polarization by activating PTEN/PI3Kγ signaling pathway^[118]^. Coculture of pancreatic cancer cells with the hypoxic cancer-derived exosomes or miR-301a-3p-upregulated macrophages was shown to facilitate the epithelial-mesenchymal transition and lung metastasis^[118]^. In epithelial ovarian cancer (EOC), hypoxic tumor-derived exosomes were shown to express a high level of miR-940, and they stimulated M2 polarization of macrophages and promoted cancer proliferation and migration^[119]^. A differential miRNA expression signature was also identified in the EOC-derived exosome under hypoxia to promote M2 polarization. miR-21-3p, miR-125b-5p, and miR-181d-5p were induced by HIF-1α and HIF-2α in the exosomes under hypoxic conditions, which regulate SOCS4/5-STAT3 signaling to stimulate M2 polarization and a malignant TME^[120]^. In melanoma, let-7a was shown to be downregulated in hypoxic cancer cells but remarkably increased in the hypoxic cancer-derived exosomes^[121]^. The exosomes carrying let-7a were found to promote a metabolic shift towards enhanced mitochondrial oxidative phosphorylation in macrophages by suppressing insulin-Akt-mTOR signaling to enhance cancer progression^[121]^.

## SUMMARY

Rapidly expanding and hypoxic tumors exploit exosomes to communicate with both cancerous and non-cancerous cells in the TME to promote cancer survival and resist immune surveillance. Hypoxia has been shown to directly induce the production of exosomes, modulate the exosome cargo sorting process, and promote exosome uptake by recipient cells. Under low oxygen tension, cancer cells are primed to glycolytic metabolism, thus inducing an acidic TME to indirectly promote intracellular transport of the exosome. Numerous regulatory molecules are involved in the regulation of exosome biology under hypoxia. More studies are warranted to fully unravel the effect of hypoxia on exosome-mediated intercellular communication within the TME.

## FUTURE PERSPECTIVES

Advances in precision oncology have led to the increasing application of tissue and liquid biopsy methods in clinical practice to facilitate treatment selection and monitoring of cancer progression. For the traditional method using tissue biopsy, limited tissue specimens are taken from the patients. They are not able to reflect the spatial and temporal heterogeneity of a primary tumor or between multiple potentially discordant metastatic lesions. In comparison with tumor tissue analysis, liquid biopsy is less invasive, and the samples can be obtained throughout disease progression. The liquid biopsy analytes include circulating tumor cells, circulating nucleic acids^[122]^, extracellular vesicles^[123]^, and other tumor-derived materials present in blood and other body fluids. Among various liquid biopsy analytes, exosomes are unique in the way that they contain not only DNA but also RNAs, ncRNAs, proteins, glycoconjugates, and lipids, thus making them more versatile biomarkers.

Currently, the exogenous hypoxic marker drug pimonidazole has been used to visualize hypoxic regions in histological sections of tumors in pathological research *in vivo*^[124]^. However, the method is invasive and involves the surgical removal of tumors for imaging. Therefore, the application of tumor-derived exosomes from biological fluid to reveal the presence of hypoxic tumors will be beneficial. A few exosome biomarkers have been shown to reflect the hypoxic status of tumors as well as the stage of tumor progression. Exosomes derived from hypoxic glioma cells were enriched with hypoxia-related mRNA and proteins (including caveolin 1, IL-8, MMPs, and PDGF)^[34]^. Importantly, patients presenting with high levels of these biomarkers were associated with worse survival^[34]^. In rectal cancer patients, low levels of miR-486-5p and miR-181a-5p but high levels of miR-30d-5p in the exosomes harvested from plasma samples are associated with hypoxic tumors and poor prognosis^[125]^. Indeed, exosomes have been used as diagnostic or prognostic tools for assessing hypoxic tumors in recent clinical trials [Table 3].

**Table 3:** Representative clinical trials exploiting exosome biomarkers to assess hypoxic tumors

**Cancer type**	**ClinicalTrials.gov Identifier**	**Aim(s) relevant to exosome biology**	**Current status**
Lung cancer	NCT04629079 (LungExoDETECT)	• To validate exosomal assays that are based on hypoxia detection as potential biomarkers for early detection • Compare exosomal analysis with the standard of care imaging	Recruiting; Started in October 2020
Lung cancer	NCT04529915	• Multicenter clinical research for early diagnosis of lung cancer using exosomes derived from blood plasma	Active; Not recruiting; Started in April 2020
Colorectal cancer	NCT04394572 (EXOSCOL01)	• To identify new diagnostic protein markers (e.g., integrins and metalloproteases) for colorectal cancer in circulating tumor exosomes	Recruiting; Started in January 2021
Clear cell renal carcinoma	NCT04053855	• To analyze urinary exosomes as a liquid biopsy tool for early diagnosis of clear cell renal cell carcinoma	Recruiting; Started in August 2019
Ovarian cancer	NCT03738319 (EOC-EXOSOME)	• To analyze the expression of miRNA and lncRNA from exosomes in blood samples by next-generation sequencing in patients with high grade serous ovarian cancer or benign gynecologic diseases	Recruiting; Started in November 2018
Melanoma	NCT02310451	• Pilot study to examine exosomes collected from the blood before and after BRAF inhibitor therapy in patients with advanced unresectable or metastatic BRAF mutation-positive melanoma • To develop an exosome-based theranostic tool for personalized care in melanoma patients	Recruiting; Started in 2016

miRNA: MicroRNA; lncRNA: long non-coding RNA.

As hypoxic tumors produce exosomes to promote tumorigenesis, the inhibition of exosome formation and secretion may be exploited as a novel strategy to suppress tumor development. In an excellent recent review, He *et al*. summarized the strategies for exosomal targeting and discussed the potential clinical applications^[126]^. Experimental reagents such as manumycin A and GW4869 were shown to inhibit exosome biogenesis and secretion from mammalian cells^[127]^. On the other hand, the Rab family of GTPases involved in exosome secretion can also be targeted to hinder exosome-mediated intercellular communication. For example, Rab5a is involved in the early step of exosome biogenesis, whereas Rab11, Rab27a, and Rab35 regulate the fusion of multivesicular bodies with the plasma membrane and exosome secretion^[128]^. Downregulation of Rab27a has been shown to inhibit exosome-dependent and -independent tumor cell growth^[128]^. Specific inhibition of sphingomyelinase (an enzyme catalyzing the formation of ceramide from sphingomyelin) has also been shown to suppress exosome biogenesis and cargo loading, thereby retarding tumor growth^[63]^.

The removal of oncogenic exosomes has been investigated as a novel therapeutic strategy for cancer therapy^[129]^. Mesoporous silica nanoparticles loaded with EGFR-targeting aptamers have been used to mop up circulating cancer-secreted EGFR+ exosomes, thus preventing their entry into the small intestine to suppress metastasis of lung cancer cells^[129]^. On the other hand, exosomes from immune cells were shown to exhibit anticancer activity^[17]^. Recently, Jiang *et al*. reported the induction of exosome production from NK cells under hypoxia^[130]^. More importantly, compared to normoxic conditions, NK cell-derived exosomes were found to express remarkably higher levels of FasL, perforin, and granzyme B in hypoxia to produce a higher NK cell cytotoxic effect^[130]^. Therefore, hypoxia-treated NK cells may be used to potentiate cancer immunotherapy.

Exosomes may also be employed as a drug delivery system for cancer therapy. Drugs or therapeutic siRNAs could be loaded into exosomes by various methods such as direct incubation, electroporation, and sonication^[131]^. Zhuang *et al*. reported the encapsulation of curcumin or an investigational STAT3 inhibitor (JSI124) in cancer cell-derived exosomes by incubating the exosomes with the drugs^[132]^. The drug-loaded exosomes were delivered to the brain for the treatment of inflammation via an intranasal route^[132]^. Besides, therapeutic siRNAs could also be loaded into the hydrophilic core of exosomes in the pharmaceutically active form^[133,134]^. Recently, Alvarez-Erviti *et al*. reported the successful delivery of siRNAs to the mouse brain using dendritic cell-derived exosomes^[135]^. Alternatively, the anticancer drug paclitaxel has been loaded indirectly into exosomes secreted from gingival mesenchymal stromal cells (MSCs) after co-culturing them with the drug^[136]^. Importantly, the exosomes derived from MSCs after priming with paclitaxel were shown to exhibit significant anticancer activity against human pancreatic cancer cells *in vitro*^[136]^. Interestingly, in a recent study examining anticancer drug delivery by exosomes, exosomes from hypoxic human breast cancer cells loaded with olaparib (a PARP inhibitor) were found to exhibit a superior uptake rate when they were co-cultured with hypoxic cancer cells^[20]^. A more detailed investigation of exosome loading and production under hypoxic conditions is advocated to further optimize exosome-mediated drug delivery.

## DECLARATIONS

### Copyright

© The Author(s) 2022.
